# Supplementary material for: Computationally inferred cell-type specific epigenome-wide DNA methylation analysis unveils distinct methylation patterns among immune cells for HIV infection in three cohorts
Source: PLoS Pathog. 2024 Mar 11;20(3):e1012063. doi: 10.1371/journal.ppat.1012063 (PMC10957090; doi:10.1371/journal.ppat.1012063)
Supplement: S1 Fig — (a) Cohort 1: VACS cell proportion. (b) Cohort 2: WIHS cell proportion. Cohort 3: GSE217633 cell proportion. Granulocytes were removed in the sequential cell-type based EWAS analyses. EWAS: Epigenome-wide Association Study; VACS: Veteran Aging Cohort Study; WIHS: Women’s Interagency HIV Study. (PDF) [file ppat.1012063.s032.pdf]

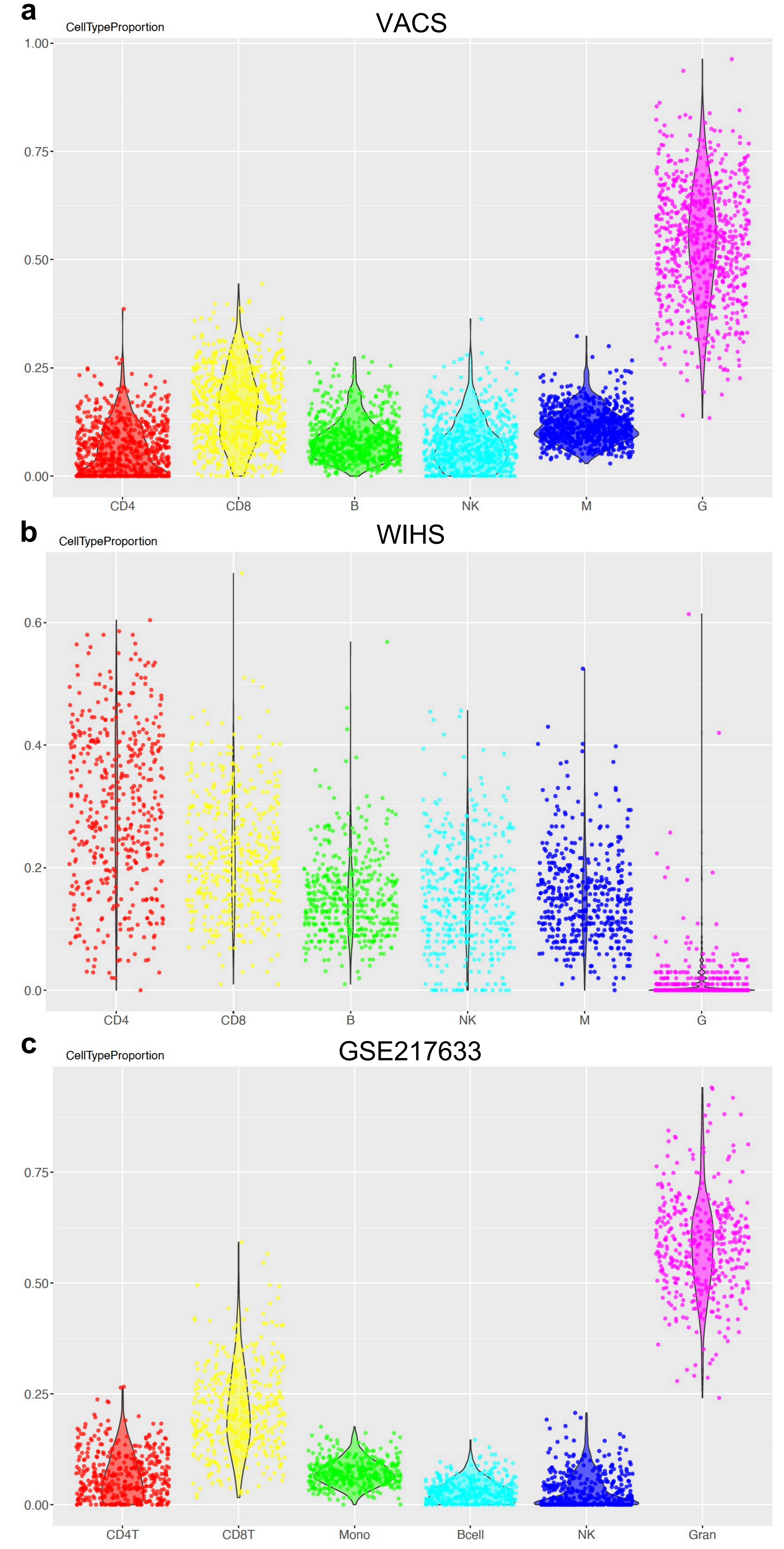

Supplemental Figure 1. Cell proportion estimation prior to TCA in both VACS and WIHS cohorts. (a) Cohort 1: VACS cell proportion. (b) Cohort 2: WIHS cell proportion. Cohort 3: GSE217633 cell proportion. Granulocytes were removed in the sequential cell-type based EWAS analyses. EWAS: Epigenome-wide Association Study; VACS: Veteran Aging Cohort Study; WIHS: Women's Interagency HIV Study.
