# Supplementary material for: Computationally inferred cell-type specific epigenome-wide DNA methylation analysis unveils distinct methylation patterns among immune cells for HIV infection in three cohorts
Source: PLoS Pathog. 2024 Mar 11;20(3):e1012063. doi: 10.1371/journal.ppat.1012063 (PMC10957090; doi:10.1371/journal.ppat.1012063)
Supplement: S2 Fig — (a) VACS: -log10(p) plots for whole blood methylation and for six individual cell type methylation. (b) WIHS: -log10(p) plots for PBMC methylation and for six individual cell type methylation. PBMC: peripheral blood mononuclear cell; TCA: Tensor Composition Analysis; VACS: Veteran Aging Cohort Study; WIHS: Women’s Interagency HIV Study. (PDF) [file ppat.1012063.s033.pdf]

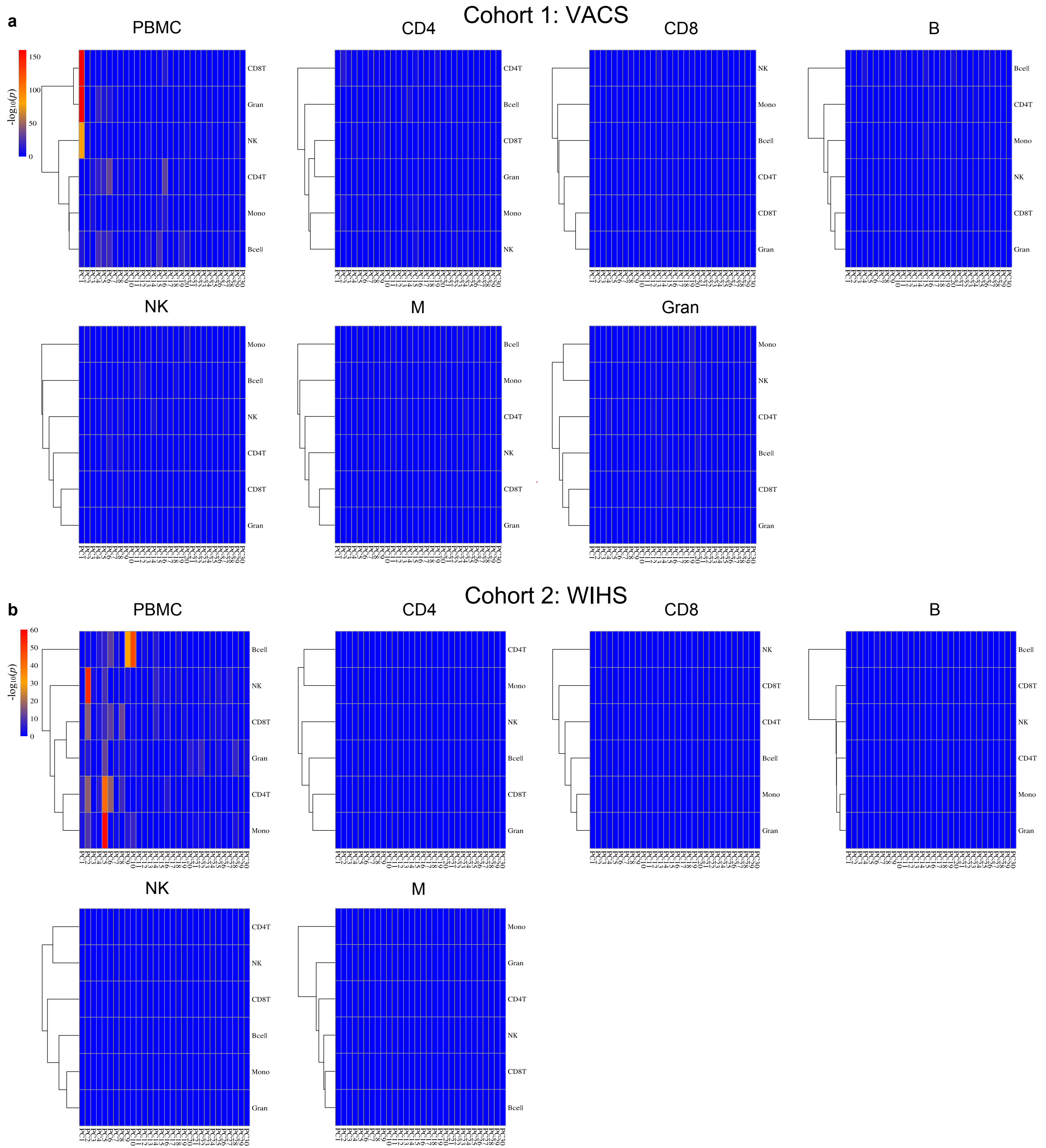

Supplemental Figure 2. Correlation analysis of top 30 Principle Components (PCs) on DNA methylation and cell type proportion in bulk cells and TCA-deconvoluted cell types from cohorts 1 and 2, for which methylation intensity data of each probe was available. (a) VACS: -$\log_{10}$(p) plots for whole blood methylation and for six individual cell type methylation. (b) WIHS: -$\log_{10}$(p) plots for PBMC methylation and for six individual cell type methylation. PBMC: peripheral blood mononuclear cell; TCA: Tensor Composition Analysis; VACS: Veteran Aging Cohort Study; WIHS: Women's Interagency HIV Study.
