# Supplementary material for: Computationally inferred cell-type specific epigenome-wide DNA methylation analysis unveils distinct methylation patterns among immune cells for HIV infection in three cohorts
Source: PLoS Pathog. 2024 Mar 11;20(3):e1012063. doi: 10.1371/journal.ppat.1012063 (PMC10957090; doi:10.1371/journal.ppat.1012063)
Supplement: S3 Fig — Comparison of methylation β-values for the top 10,000 most variable CpG sites between the deconvoluted and the directly measured methylation. β-values for each cell type were compared between three cell types [CD4+ T-cells, CD8+ T-cells, and monocytes (CD16+)]. MC: methylation capture sequencing; TCA: Tensor Composition Analysis. (PDF) [file ppat.1012063.s034.pdf]

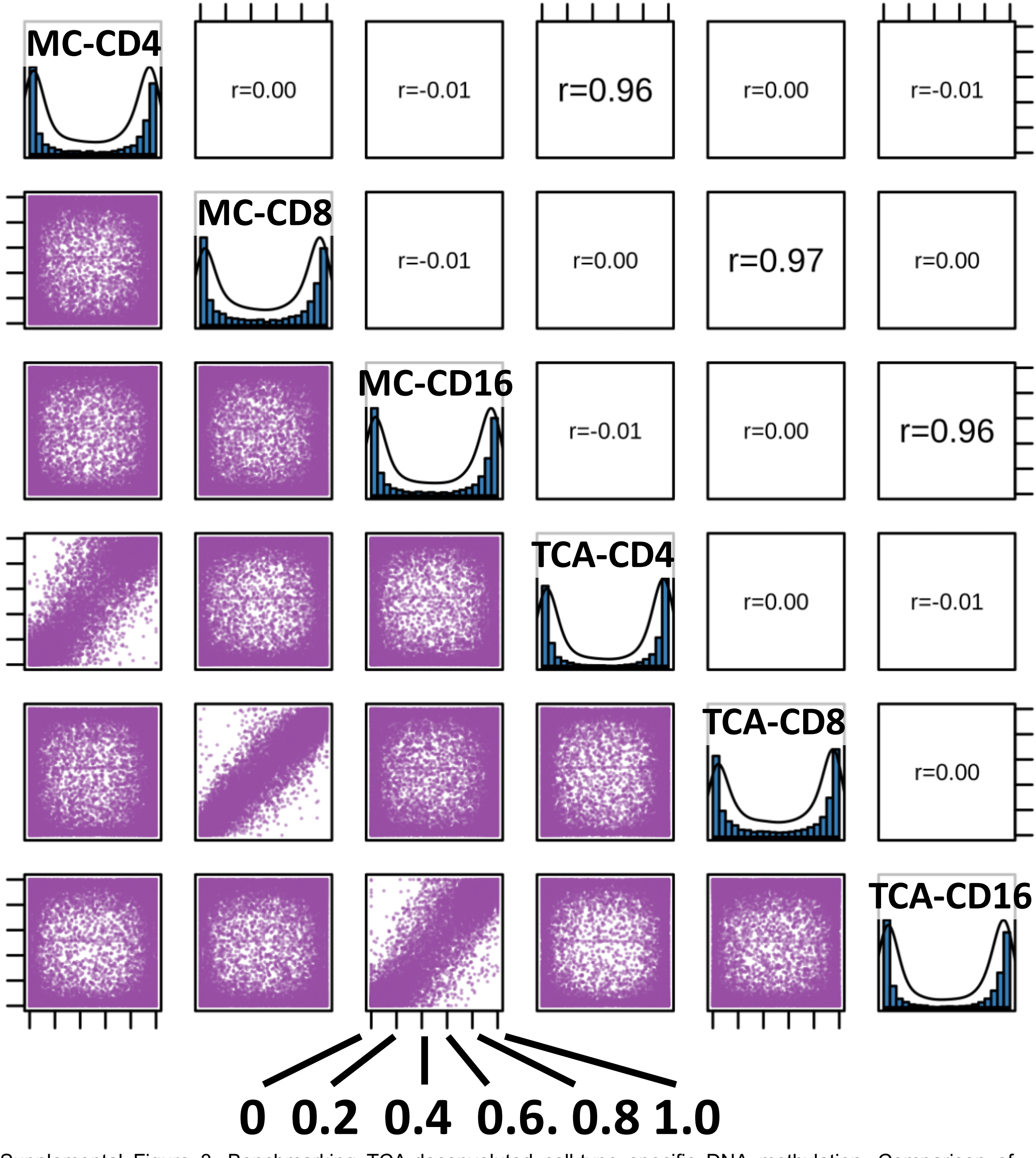

Supplemental Figure 3. Benchmarking TCA-deconvoluted cell-type specific DNA methylation. Comparison of methylation β-values for the top 10,000 most variable CpG sites between the deconvoluted and the directly measured methylation. β-values for each cell type were compared between three cell types [CD4+ T-cells, CD8+ T-cells, and monocytes (CD16+)]. MC: methylation capture sequencing; TCA: Tensor Composition Analysis.
