# Supplementary material for: Computationally inferred cell-type specific epigenome-wide DNA methylation analysis unveils distinct methylation patterns among immune cells for HIV infection in three cohorts
Source: PLoS Pathog. 2024 Mar 11;20(3):e1012063. doi: 10.1371/journal.ppat.1012063 (PMC10957090; doi:10.1371/journal.ppat.1012063)
Supplement: S4 Fig — (a) Cohort 1: VACS, EWAS on HIV infection in whole blood. (b) Cohort 1: VACS, EWAS on HIV infection in 5 individual cell types. (c) Cohort 2: WIHS, EWAS on HIV infection in PBMCs. (d) Cohort 2: WIHS, EWAS on HIV infection in 5 individual cell types. (e) Cohort 3: GSE217633, EWAS on HIV infection in PBMCs. (f) Cohort 3: GSE217633, EWAS on HIV infection in 5 individual cell types. EWAS: Epigenome-wide Association Study; VACS: Veteran Aging Cohort Study; WIHS: Women’s Interagency HIV Study. (PDF) [file ppat.1012063.s035.pdf]

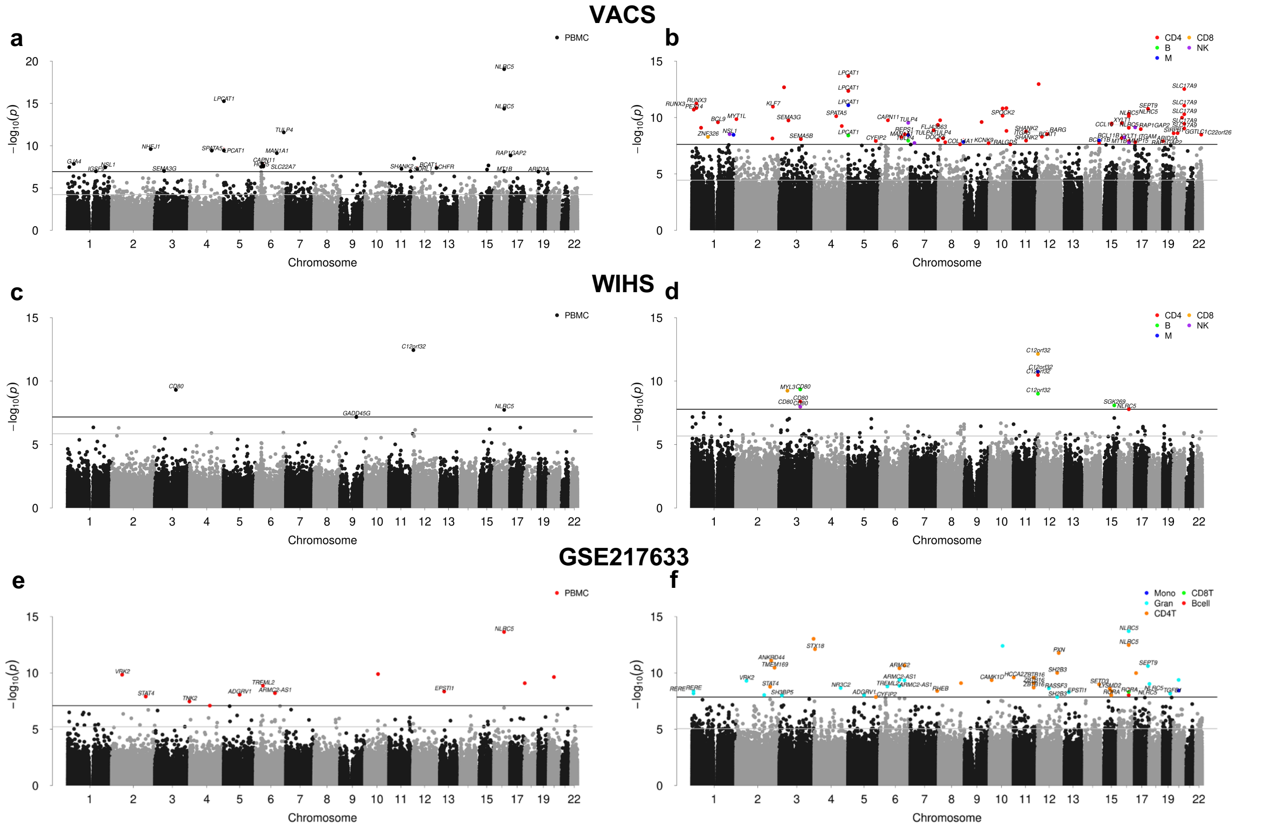


Supplemental Figure 4. Summary of EWAS prior to and following computational deconvolution in each cohort. (a) Cohort 1: VACS, EWAS on HIV infection in whole blood. (b) Cohort 1: VACS, EWAS on HIV infection in 5 individual cell types. (c) Cohort 2: WIHS, EWAS on HIV infection in PBMCs. (d) Cohort 2: WIHS, EWAS on HIV infection in 5 individual cell types. (e) Cohort 3: GSE217633, EWAS on HIV infection in PBMCs. (f) Cohort 3: GSE217633, EWAS on HIV infection in 5 individual cell types. EWAS: Epigenome-wide Association Study; VACS: Veteran Aging Cohort Study; WIHS: Women's Interagency HIV Study.
