# Supplementary material for: Computationally inferred cell-type specific epigenome-wide DNA methylation analysis unveils distinct methylation patterns among immune cells for HIV infection in three cohorts
Source: PLoS Pathog. 2024 Mar 11;20(3):e1012063. doi: 10.1371/journal.ppat.1012063 (PMC10957090; doi:10.1371/journal.ppat.1012063)
Supplement: S5 Fig — EWAS: Epigenome-wide Association Study; PBMC: peripheral blood mononuclear cell. (PDF) [file ppat.1012063.s036.pdf]

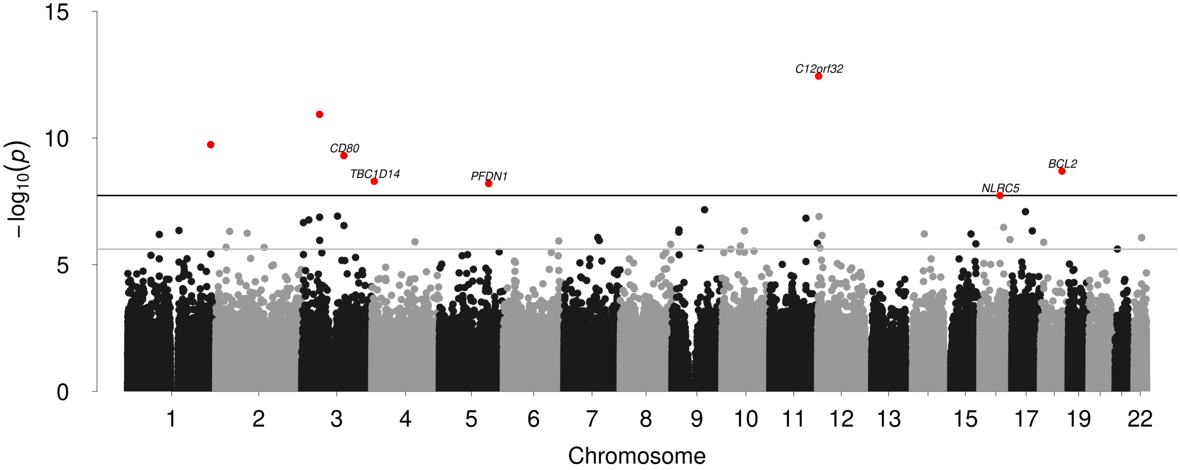

Supplemental Figure 5. EWAS on HIV in cohort 2 PBMCs using all ~870K CpG sites in the EPIC array. EWAS: Epigenome-wide Association Study; PBMC: peripheral blood mononuclear cell.
