# Supplementary material for: Computationally inferred cell-type specific epigenome-wide DNA methylation analysis unveils distinct methylation patterns among immune cells for HIV infection in three cohorts
Source: PLoS Pathog. 2024 Mar 11;20(3):e1012063. doi: 10.1371/journal.ppat.1012063 (PMC10957090; doi:10.1371/journal.ppat.1012063)
Supplement: S7 Fig — DMP: Differential Methylation Position. (PDF) [file ppat.1012063.s038.pdf]

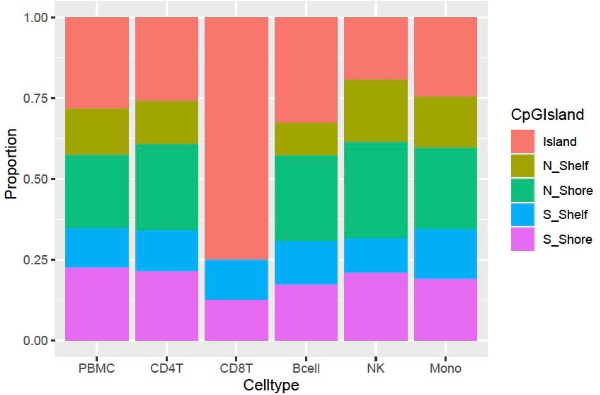

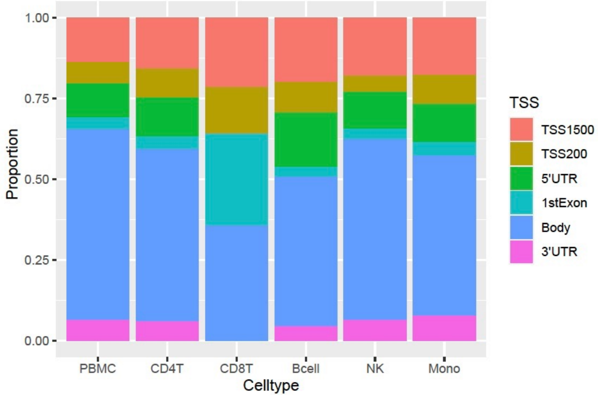

Supplemental Figure 7. Characterization of epigenome-wide significant DMP from cell-type level meta-epigenome-wide association analysis. DMP: Differential Methylation Position.
