# Supplementary material for: Computationally inferred cell-type specific epigenome-wide DNA methylation analysis unveils distinct methylation patterns among immune cells for HIV infection in three cohorts
Source: PLoS Pathog. 2024 Mar 11;20(3):e1012063. doi: 10.1371/journal.ppat.1012063 (PMC10957090; doi:10.1371/journal.ppat.1012063)
Supplement: S8 Fig — (PDF) [file ppat.1012063.s039.pdf]

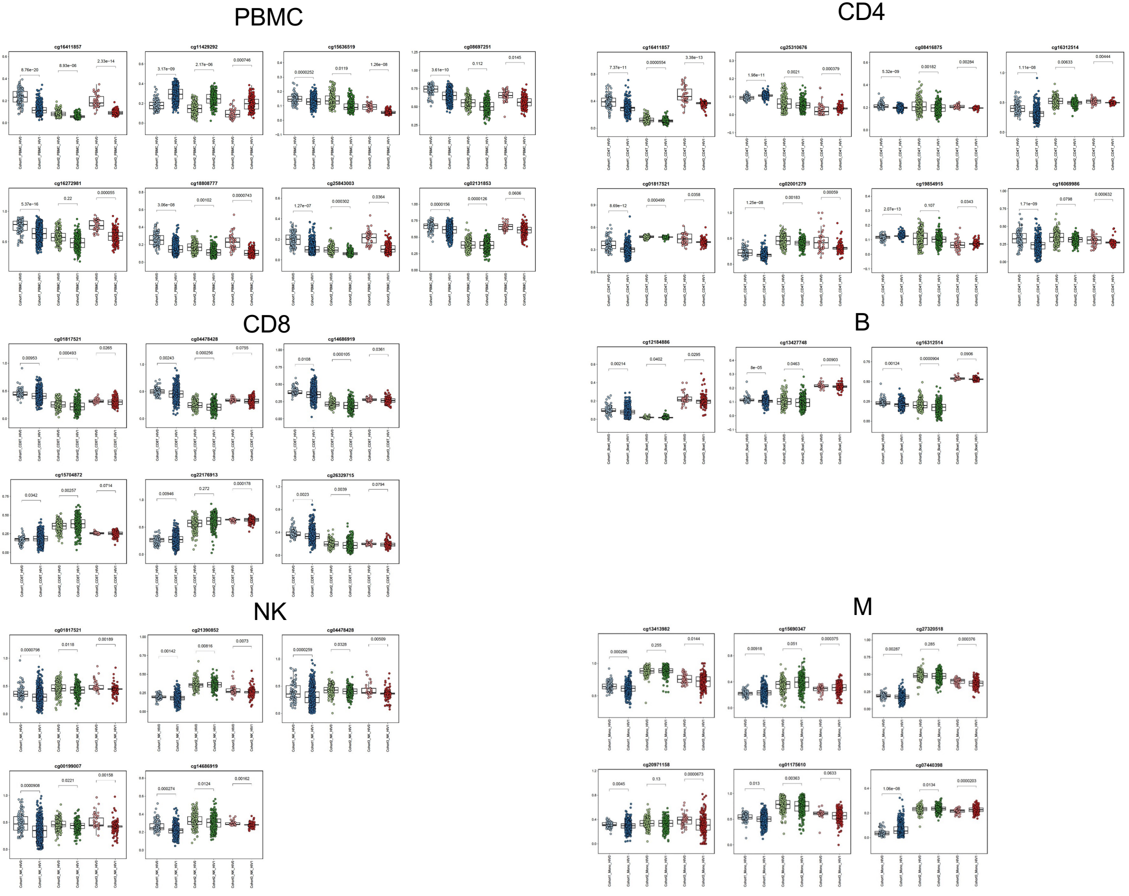

Supplemental Figure 8. Beta values in HIV+ and HIV- groups among three cohorts for the top meta-significant CpG sites in which beta value differed at least 5% between HIV+ and HIV- in at least two of three cohorts.
