# Supplementary material for: Computationally inferred cell-type specific epigenome-wide DNA methylation analysis unveils distinct methylation patterns among immune cells for HIV infection in three cohorts
Source: PLoS Pathog. 2024 Mar 11;20(3):e1012063. doi: 10.1371/journal.ppat.1012063 (PMC10957090; doi:10.1371/journal.ppat.1012063)
Supplement: S9 Fig — (PDF) [file ppat.1012063.s040.pdf]

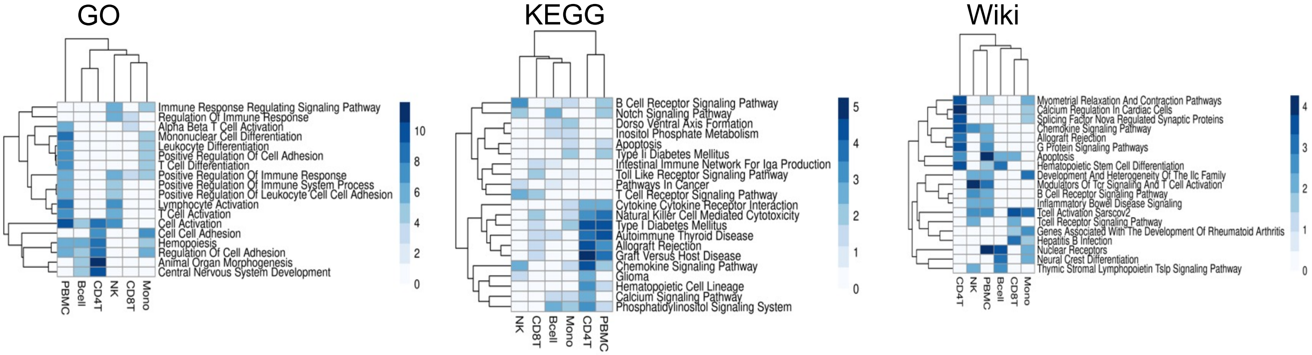

Supplemental Figure 9. GO (Gene Ontology), KEGG (Kyoto Encyclopedia of Genes and Genomes), and Wiki pathway analysis for each cell type.
