# Supplementary material for: Computationally inferred cell-type specific epigenome-wide DNA methylation analysis unveils distinct methylation patterns among immune cells for HIV infection in three cohorts
Source: PLoS Pathog. 2024 Mar 11;20(3):e1012063. doi: 10.1371/journal.ppat.1012063 (PMC10957090; doi:10.1371/journal.ppat.1012063)
Supplement: Attachment — Submitted filename: Response to Reviewers.pdf [file ppat.1012063.s041.pdf]

Ke Xu, M.D. Ph.D.
Professor of Psychiatry
Yale University School of Medicine

VA Connecticut Healthcare System
950 Campbell Ave. Mailstop 116A
West Haven, CT 06516

Phone: (203) 932-5711, ext. 7430
Fax: (203) 937-4860
E-mail: ke.xu@yale.edu

Prof. Kasturi Haldar,
Editor-in-Chief
PLOS Pathogens

RE: Resubmission PPATHOGENS-D-23-01741 "**Computationally Inferred Cell-Type Specific Epigenome-Wide DNA Methylation Analysis Unveils Distinct Methylation Patterns among Immune Cells for HIV Infection in Three Cohorts**" by Zhang et al.

Dear Prof. Halder,

We appreciate the reviewer's constructive comments and the opportunity to resubmit the manuscript. To address the reviewers' concerns, we have made significant changes in this revision. The major changes include 1) a revised epigenome-wide association (EWA) analysis on HIV infection in cohort 2 using all CpG sites on the EPIC array (870K), the results of which both confirmed our previous analysis with only 450K and EPIC common CpG sites, as well as identified additional significant CpG sites associated with HIV infection; 2) a partial replication and interpretation of previously reported significant CpG sites for HIV infection in Cohort 3, analyzing Pre-ART versus HIV-negative samples; 3) a new EWA analysis for post- and pre-ART samples, leveraging the data from post-ART samples; 4) an expanded discussion on previous EWAS findings on HIV infection with a focus on cell-type specific changes; 5) a discussion of the different computational deconvolution methods available. We believe that the quality of the revised manuscript has been markedly improved and will enhance the message for the journal audience.

We address the reviewer's comments point-by-point in the response to reviewers.

Sincerely,

Ke Xu, MD, PhD and Bradley Aouizerat, PhD

Response to Reviewer(s)

We appreciate the reviewer's constructive comments. To address the reviewers' concerns, we have made significant changes in this revision. The major changes include 1) a revised epigenome-wide association (EWA) analysis on HIV infection in cohort 2 using all CpG sites on the EPIC array (870K), the results of which both confirmed our previous analysis with only 450K and EPIC common CpG sites, as well as identified additional significant CpG sites associated with HIV infection; 2) a partial replication and interpretation of previously reported significant CpG sites for HIV infection in Cohort 3, analyzing Pre-ART versus HIV-negative samples; 3) a new EWA analysis for post- and pre-ART samples, leveraging the data from post-ART samples; 4) an expanded discussion on previous EWAS findings on HIV infection with a focus on cell-type specific changes; 5) a discussion of the different computational deconvolution methods available. We believe that the quality of the revised manuscript has been markedly improved and will enhance the message for the journal audience.

We address the reviewer's comments point-by-point as below:

Reviewer #1: The manuscript by Zhang et al. focuses on cell-type specific epigenome-wide DNA methylation analysis among immune cells for HIV infection in three cohorts. The paper aims to identify differentially methylated CpG sites for HIV infection in immune cell types including CD4 T cells, CD8 T cells, B cells, NK cells, monocytes, and granulocytes which is a strength.

The methods applies a computational deconvolution method to perform a cell-type based EWAS which is a weakness since the actual cell-type specific signal is not being directly assessed as stated in the main aim of the paper.

1. The sample size total is 1,382 samples which is a strength and uses a widely utilized array-based method to assay DNA methylation states. The approach also analyzed the data in each cohort separately and then combined in a meta-EWAS. Overall, the study is significant for the field as it highlights the potential cell-type specific differences in epigenetic signals that are relevant for HIV infection.

<u>**Response:** We appreciate the encouraging comment.</u>

2. The major weakness is that the title and presentation of the approach should clarify that the cell-type specificity was bioinformatically inferred. I would advise to change to title to "Inferred cell-type specific..." and modify the text to reflect the inferred nature of the analyses for cell type-specificity. There also should be a discussion of how the deconvolution algorithm utilized may not reflect immune cell composition changes observed in people living with HIV.

<u>**Response:** Agreed. The title and the text are modified to "inferred cell-type specific" throughout the manuscript. The revised manuscript is entitled "Computationally Inferred Cell-Type Specific Epigenome-Wide DNA Methylation Analysis Unveils Distinct Methylation Patterns among Immune Cells for HIV Infection in Three Cohorts."</u>

Additionally, we discussed the difference in cell-type proportions between the algorithm inferred proportion and the immune cell compositions among PLWH. Please also see the below response to overall comment #3.

3. Reviewer #1: The results section states that many cell-type specific significant CpG sites were associated with HIV infection in each cohort. Line 35/36 is vague and doesn't state how many sites. How many sites overlapped or were concordant among the three cohorts?

Response: The meta-analysis showed a total of 2,021 cell-type unique and significant CpG sites for five cell types. The concordance rates of significant CpG sites among three cohorts were 98.6% (1,982/ 2,010) in CD4+ T cells, 100% in CD8+ T cells, 96% (273/284) in B cells, 96.7% (330/341) in NK cells, 97.5% (279/286) in monocytes. We added the concordance of significant CpG sites for each cell type in **Supplemental Tables S24-S29**. Lines 36-38 were revised to: "The meta-analysis unveiled a total of 2,021 cell-type unique significant CpG sites for five cell types. Among these inferred cell-type unique CpG sites, the concordance rate in the three cohorts ranged from 96% to 100% in each cell type."

4. The statement in Line 49 of the conclusion about pathogen-induced epigenetic oncogenicity is weakly supported by the way the manuscript is structured and the results. Just because enrichment in that category was found doesn't specify that over the other enriched categories.

Response: We agree with the reviewer and therefore deleted the "pathogen-induced epigenetic oncogenicity" in the conclusion.

5. In the introduction Line 101 and 102 is not completely correct as prior studies have examined cell sorted immune cell subsets and examined differences in HIV.

Response: We changed the sentence to "HIV-1-induced alteration to the epigenomes of specific immune cell types have not been fully understood".

6. This is correct in lines 108 and 109. Please provide references.

Response: References were added.

7. The Veterans Aging Cohort Study, Women's Interagency HIV study, and public dataset GSE217633 were used as datasets. Were the Cohort 1 VACS DNA methylation data previously published or deposited in GEO? Please clarify in the Methods.

Response: Yes, we have published and deposited the DNA methylation data from cohort 1 (VACS) in GSE107080 and GSE107082, which is clarified in the Methods now.

8. A weakness is the use of data from cohort 1 that was from an older 450k array. This limits the analysis by removed over 400,000 CpGs sites that are unique to the EPIC arrays. These sites are likely more informative as they fall in gene regulatory regions.

Response: We appreciate this constructive comment. We re-analyzed the EWAS while including all CpG sites in the EPIC array for cohort 2. Please see the response below to **#12**.

9. Results: The section about benchmarking deconvoluted DNA methylome data using capture bisulfite sequencing in CD4, CD8, and monocytes should be moved to supplemental or included in the Methods section. The figure 2 doesn't add anything significant to the results.

Response: We have moved the benchmarking section to the Methods, and Figure 2 was moved to Supplemental Figures.

10. Cohort 1's analyses should clarify in text that this used the 450K data only.

Response: We clarified the 450K array in the Results and the Methods sections.

11. Please show plots of the top CpG sites based on the beta values in supplemental.

Response: Yes, we have presented beta values in groups with and without HIV among three cohorts for the meta-significant CpG sites in major cell types as examples (**Supplemental Figure 8**), in which beta value differed at least 5% between those with and without HIV in at least two of three cohorts.

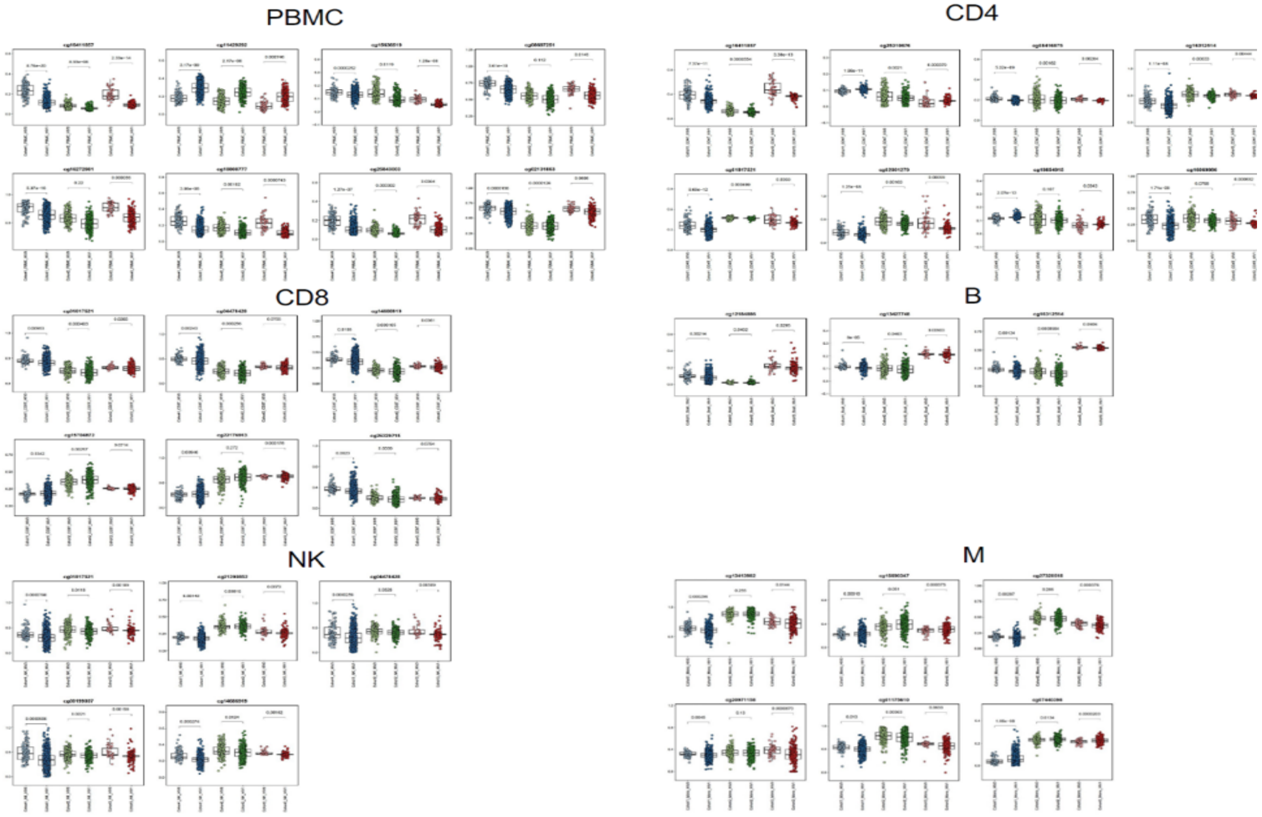

12. For Cohort 2, what would the data look like if you used all the data for the methylation EPIC arrays? Does the frequency of CpGs increase or is there a similar number? Are the genes and

pathways different? How much does the interpretation change since only the matching 450K probes are used?

Response: We appreciate this constructive comment. We performed an EWAS in bulk PBMCs using all CpG sites in the EPIC array and the same covariables in cohort 2. We identified 47 significant CpG sites with FDR<0.05. Compared to the results from the EWAS including only common CpG sites in the 450K array, we found **34** more significant CpG sites, (see **Supplemental Figure 5 and Supplemental Table 10**, a new table). Importantly, all 13 previously significant CpG sites were also significant in the EPIC-EWAS with slightly increased p values. For example, the FDR value for the top CpG, cg12051710 in C12orf32, was 1,47E-07 in 450K-EWAS but was 3.03E-07 in EPIC-EWAS. The additional significant CpG sites were located in 32 genes such as *BCL2, PFDN1, ITPR1*.

Gene set enrichment analysis (GSEA) showed that HALLMARK_INTERFERON_GAMMA_RESPONSE was significant (FDR=0.03), which is consistent with the results in the meta-GSEA (FDR=0.0004). Together, the additional analysis added more evidence supporting the findings in other cohorts and did not change our interpretation of the findings.

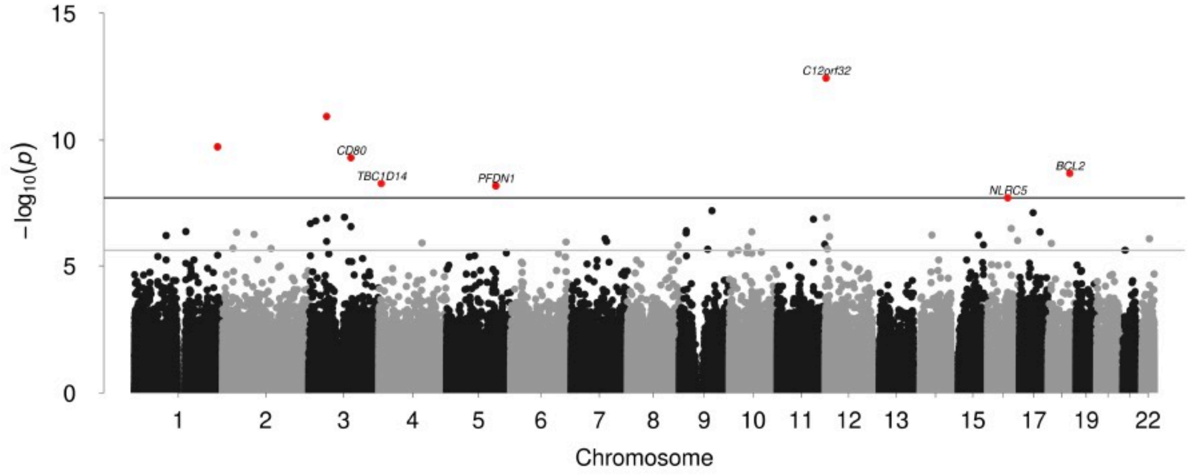

Supplemental Figure 5. EWAS on HIV in cohort 2 PBMCs using all ~870K CpG sites in the EPIC array. EWAS: Epigenome-wide Association Study; PBMC: peripheral blood mononuclear cell.

12. In the replication cohort lines 368-370, what was the percentage of loci that were replicated in whole blood? The dataset should also be utilized to replicate the post-ART time points. This would be a more relevant validation and comparison that should be included from that dataset.

Response: In the EWAS for pre-ART versus those without HIV, we identified 75 CpG sites located in 49 genes, which was fewer than the previous report. Compared to the originally reported study by Esteban-Cantos *et al.*, the fewer loci in our EWAS are likely due to the use of a more stringent a two-step regression model to comprehensively remove global and specific confounding factors compared to the original paper. We adapted the incorporation of the Control Probe Adjustment and reduction of global CORrelation (CPACOR) pipeline proposed by Lehne *et al.*[1] Only residual methylation of each CpG was applied to the model. Thus, the inflation in our EWAS is likely lower and the results are likely less spurious. Among 75 CpG sites, 39 CpG sites

were at the same loci as the original paper. At the gene level, 28 out of 49 genes were consistent with the previous report.

To replicate the post-ART time point, we performed EWAS for pre-ART versus post-ART and post-ART versus those without HIV. For the post-ART versus pre-ART EWAS, **807** CpG sites reached epigenome-wide significant (FDR<0.05) (**Supplemental Table 17, Supplemental Figure 6**). Among the significant CpG sites, 183 CpG sites were identical with the originally reported study by Esteban-Cantos *et al.* Consistent with the results from pre-ART versus without HIV, the different significant CpG sites between our analysis and Esteban-Cantos *et al.* was likely due to two different analytical models.

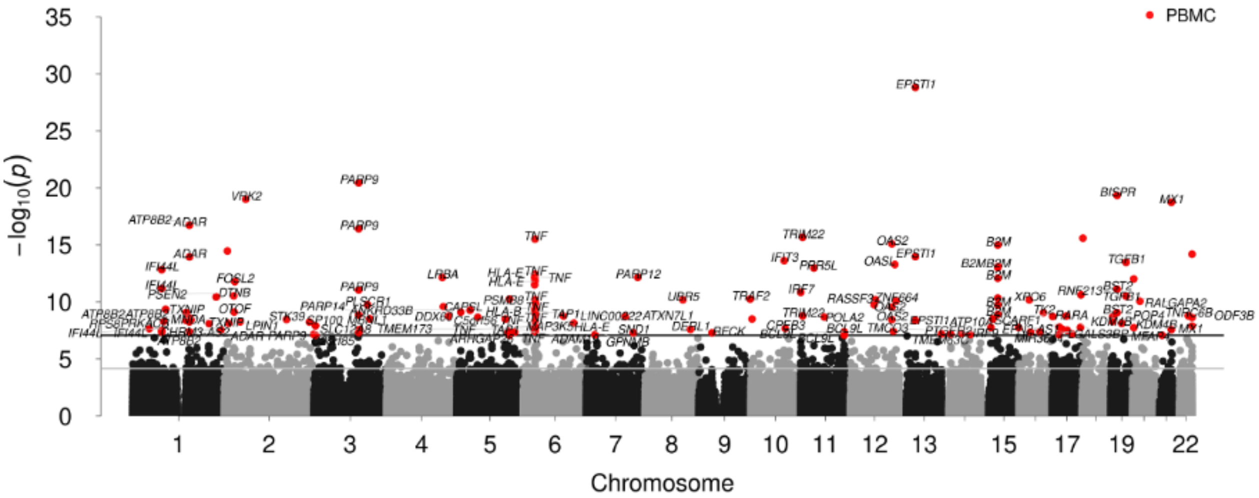

Supplemental Figure 6. Epigenome-wide association analysis on pre-ART versus post-ART

13. Line 413 please list the examples. Lines 420-423 please provide statistics for the region enrichment analyses and show a plot or supplemental figure.

Response: Several top significant DMPs encode transcription factors. For example, in meta-EWAS of CD4+ T cell, cg00676801 on *STAT1* was hypomethylated in HIV-infection (meta-FDR: 3.39E-07). STAT1 is a transcription factor involved in upregulating genes due to a signal by interferons, cytokines, and growth factors. cg05573412 on *PRDM16*, encoding a zinc finger transcription factor that plays a role in the regulation of developmental processes, was hypermethylated (meta-FDR: 2.06E-06). cg15498134 on *RUNX3*, a gene involved in lineage-specific gene expression in a variety of cells, was hypomethylated in HIV infected samples (meta-FDR: 9.74E-06).

We added statistics on the region enrichment analyses in lines 449-452. "Regional enrichment analysis showed that DMPs in the promoter region were significant in CD4+ T cells (χ^2=5.01; p=0.025), but not significant in other cell types." We present the proportion of CpG regions in **Supplemental Figure 7** (see above).

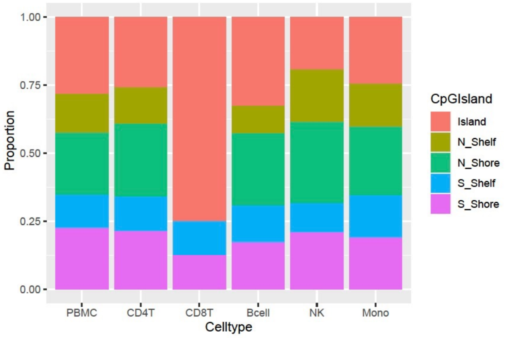

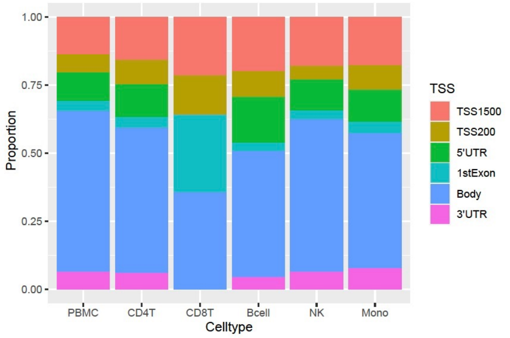

15. The results section on line 424 should link to the cohort metadata. Do any of the participants have cancer?

Response: Unfortunately, data about cancer diagnoses were not available to us.

16. The section on HIV-associated DMPs analyses should specify the database used and potentially consider using multiple databases to validate the results. Sometimes these analyses are database biased since the majority have focused on cancer.

Response: We agree with the reviewer. The datasets we used for DMP enrichment originated from the Molecular Signature Database. “Hallmark gene sets summarize and represent specific well-defined biological states or processes and display coherent expression. These gene sets were generated by a computational methodology based on identifying gene set overlaps and retaining genes that display coordinate expression. The hallmarks reduce noise and redundancy and provide a better delineated biological space for GSEA. We refer to the original overlapping gene sets, from which a hallmark is derived, as its 'founder' sets. Hallmark gene set pages provide links to the corresponding founder sets for more in-depth exploration. In addition, hallmark gene set pages include links to microarray data that served for refining and validation of the hallmark signatures.”

As the reviewer suggested, we re-analyzed the cell-type based GSEA using meta-significant DMPs in three additional databases: Gene Ontology (GO), KEGG: Kyoto Encyclopedia of Genes and Genomes, and Wiki pathways. **Supplemental Figure 9** shows the top 15 GO and KEGG pathways in each cell type. Overall, immune response, autoimmune disease, and cancer pathways were among top 15 pathways.

GO: Biological Position

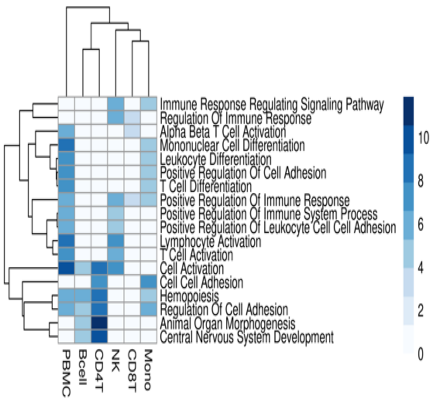

KEGG

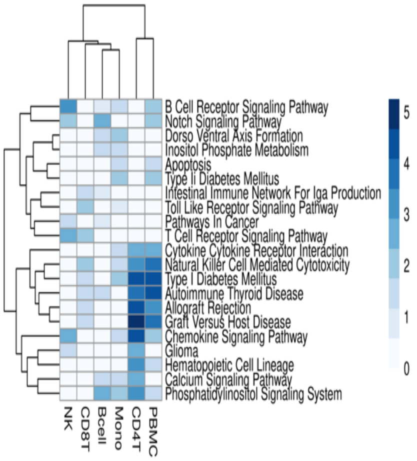

Wiki

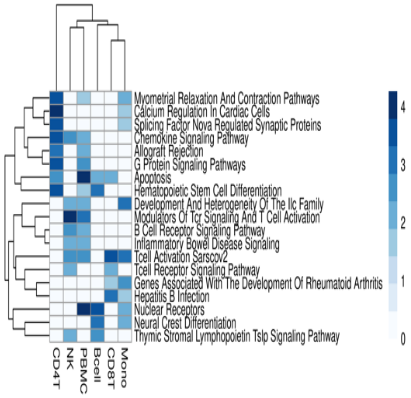

17. The discussion should be lengthened to better review prior studies and to provide a more thorough discussion of the cell-type specific implications of these findings.

Response: We discussed prior studies of DNA methylation on HIV infection here and in lines 498-541.

“Because *NLRC5* is a transcription factor regulating the major histocompatibility complex (MHC), and methylation of *NLRC5* has been consistently reported as having an association with HIV infection[2-4], several studies have examined differential methylation patterns on chromosome 6 MHC region for HIV infection. Shiau et al identified 28 differentially methylated regions on chromosome 6 for children receiving antiretroviral therapy (ART)[5]. Gross et al reported significant hypomethylation of Chromosome 6 around HLA region for those with HIV compared to those without HIV in CD4+ T cells and PBMCs[6]. Our results are consistent with these findings of hypomethylation of HLA genes with HIV infection in PBMCs and CD4+ T cells. For example, a meta-EWAS in CD4+ T cells showed that five CpG sites on HLA-F (cg23892836, cg12588917, cg09296453, cg24351901, cg15331332), two CpG sites on HLA-E (cg14461571 and cg03725115), and one CpG (cg11187245) were all hypomethylated in those with HIV versus those without HIV across three cohorts. Together, these studies provide insights on the alteration of DNA methylation in MHC influencing immune regulation in HIV-1 pathogenesis.

Methylation changes in other immune genes and pathways beyond MHC are also linked with HIV status, ART, and clinical outcomes. DMPs for PWH pre-ART versus those without HIV were enriched in the pathways involving in immune function such as regulation of immune system processes, immune response, and response to cytokines[2]. In our 3-cohort meta-EWAS, those pathways were also significantly associated with HIV infection (GOBP_POSITIVE_REGULATION_OF_IMMUNE_SYSTEM_PROCESS: whole blood/PBMC q value = 0.000270779; CD4+ T cell: q= 0.00288; GOBP_POSITIVE_REGULATION_OF_IMMUNE_RESPONSE, PBMC/whole blood: q value = 0.00027; CD4+ T: q=0.01) GOBP_POSITIVE_REGULATION_OF_CYTOKINE_PRODUCTION, PBMC/whole blood: q value = 0.000282487).

More importantly, prior research has demonstrated HIV-1-induced alterations in landscape DNA methylation within monocytes and CD4 T cells during the early stages of HIV infection. In a study conducted by Corley et al[7], 22 participants at various Fiebig stages were examined. The median duration of exposure of HIV-1 to DNA methylation profiling was 17.5 days. In this acute phase of HIV infection, Corley and colleagues identified 22,697 DMPs in monocytes and 294 DMPs in CD4+ T cells[7], suggesting significant differences in DNA methylation modifications between cell types. Our results provide further evidence to support this conclusion.

Interestingly, in chronic HIV infection, we observed more DMPs in CD4+ T cells compared to monocytes. Furthermore, Corley et al observed a higher count of hypomethylated DMPs compared to hypermethylated DMPs in both monocytes and CD4+ T cells during acute HIV infection. These DMPs were associated with enriched pathways involved in antiviral defense mechanisms and cell division. Conversely, our findings revealed a slightly greater number of hypermethylated DMPs than hypomethylated ones. Genes related to these DMPs were enriched in pathways associated with inflammatory functions. CD4+ T cells serve as a major host for HIV-1 latent reservoirs. Notably, DNA methylation in both host and HIV-1 epigenomes plays a critical role in establishing and maintaining HIV-1 reservoirs. It is plausible that alterations in DNA methylation within chronically infected CD4+ T cells may contribute to HIV-1 survival, expansion, and latency persistence. Together, these results show a distinct methylation profile between monocytes and CD4+ T cells in acute versus chronic stages of HIV-1 infection.

Overall the following three major issues should be addressed:

1. Presentation of beta values for the DMP examples to show biological relevance of the identified loci.

Response: Please see the response to comment #11.

2. Reanalysis and comparison using the removed 400,000 sites from the EPIC array for COhort 2 and inclusion of Cohort 3's post-ART data for validation.

Response: Please see the response to comment #12.

3. A comparison and discussion of how the cell-type deconvolution algorithms and datasets changes the results.

Response: Please see lines 583-598.

In recent years, several cell type deconvolution algorithms have been developed for identifying disease-associated CpG sites at the cell type resolution[8-11]. The algorithms include reference-based methods, such as CIBERSORT (CBS)[11] and EpiDISH[12] and reference-free methods such as TCA. The reference-based methods use the known cell-type methylation derived from sorted cell types, while reference-free methods are based on unsupervised methods to infer putative

cell proportion. How these algorithms can be carried out for cell-type specific EWAS with real-world methylation data is unclear.

Two methods have been tested for multiple EWAS: CellDMC and TCA[13,14]. CellDMC, taking cell type DNA methylation derived from EpiDISH, incorporates an interaction term of phenotype and estimated cell-type proportion in the linear model and enables to resolve variation in effect-size as a function of cell-type abundance in a cell-type specific manner. On the other hand, TCA is to use a tensor composition analysis to learn cell-type-specific DNA methylation from a typical two-dimensional bulk data (samples by methylation sites). Conceptually, TCA considers that individuals not only differ on cell type proportion but differ on methylation within a cell type. We chose to apply TCA method for this study because TCA appears having greater power than CellDMC and because TCA deconvolute methylation value at each CpG that is helpful for benchmarking using real methylation data in sorted cell types and biological interpretations of the results.

Part III – Minor Issues: Editorial and Data Presentation Modifications
Please use this section for editorial suggestions as well as relatively minor modifications of existing data that would enhance clarity.

Reviewer #1: Figure 3 C is missing label for granulocytes. Please clarify in Lines 342 that the dataset was filtered to remove 400,000 sites to match the VACS cohort. Line 348 please provide the p values.

Response: We have corrected the errors.

References:

1. Lehne, B. *et al.* A coherent approach for analysis of the Illumina HumanMethylation450 BeadChip improves data quality and performance in epigenome-wide association studies. *Genome Biol* **16**, 37 (2015).
2. Esteban-Cantos, A. *et al.* Effect of HIV infection and antiretroviral therapy initiation on genome-wide DNA methylation patterns. *EBioMedicine* **88**, 104434 (2023).
3. Zhang, X. *et al.* Epigenome-wide differential DNA methylation between HIV-infected and uninfected individuals. *Epigenetics* **11**, 750-760 (2016).
4. Shu, C. *et al.* Epigenome-wide association scan identifies methylation sites associated with HIV infection. *Epigenomics* **12**, 1917-1927 (2020).
5. Shiau, S. *et al.* Distinct epigenetic profiles in children with perinatally-acquired HIV on antiretroviral therapy. *Sci Rep* **9**, 10495 (2019).
6. Gross, A.M. *et al.* Methylome-wide Analysis of Chronic HIV Infection Reveals Five-Year Increase in Biological Age and Epigenetic Targeting of HLA. *Mol Cell* **62**, 157-168 (2016).

7. Corley, M.J. *et al.* Abrupt and altered cell-type specific DNA methylation profiles in blood during acute HIV infection persists despite prompt initiation of ART. *PLoS Pathog* **17**, e1009785 (2021).
8. Titus, A.J., Gallimore, R.M., Salas, L.A. & Christensen, B.C. Cell-type deconvolution from DNA methylation: a review of recent applications. *Hum Mol Genet* **26**, R216-R224 (2017).
9. Rahmani, E. *et al.* Cell-type-specific resolution epigenetics without the need for cell sorting or single-cell biology. *Nature Communications* **10**, 3417 (2019).
10. Zheng, S.C., Breeze, C.E., Beck, S. & Teschendorff, A.E. Identification of differentially methylated cell types in epigenome-wide association studies. *Nat Methods* **15**, 1059-1066 (2018).
11. Newman, A.M. *et al.* Robust enumeration of cell subsets from tissue expression profiles. *Nat Methods* **12**, 453-7 (2015).
12. Teschendorff, A.E., Breeze, C.E., Zheng, S.C. & Beck, S. A comparison of reference-based algorithms for correcting cell-type heterogeneity in Epigenome-Wide Association Studies. *BMC Bioinformatics* **18**, 105 (2017).
13. Haftorn, K.L. *et al.* Nucleated red blood cells explain most of the association between DNA methylation and gestational age. *Commun Biol* **6**, 224 (2023).
14. Tseng, C.C. *et al.* Cell lineage-specific methylome and genome alterations in gout. *Aging (Albany NY)* **13**, 3843-3865 (2021).
